# Polymer Nanoparticle-Based Photodynamic Therapy Combined with Immunotherapy for Solid Tumor Treatment

**DOI:** 10.3390/cimb48030281

**Published:** 2026-03-05

**Authors:** Jieling Lao, Qiuting Ye, Shijie Fan, Zhengqing Cheng, Pan Wu

**Affiliations:** 1 State Key Laboratory of Targeting Oncology, National Center for International Research of Bio-Targeting Theranostics, Guangxi Key Laboratory of Bio-Targeting Theranostics, Collaborative Innovation Center for Targeting Tumor Diagnosis and Therapy, Guangxi Talent Highland of Major New Drugs Innovation and Development, Targeting Theranostics Research Center of Guangxi Higher Education, Guangxi Medical University, Nanning 530021, China; 202321622@sr.gxmu.edu.cn (J.L.); ye_qiuting@163.com (Q.Y.); 13807846373@163.com (S.F.); zhengqing1108@163.com (Z.C.); 2 Pharmaceutical College, Guangxi Medical University, Nanning 530021, China

**Keywords:** polymer nanoparticles, photodynamic therapy, immunotherapy, tumor treatment

## Abstract

Polymer nanoparticles have been widely studied for tumor treatment due to their excellent biocompatibility, structural diversity, and multi-functionality. Among their various applications, combining polymer-based photosensitizers with photodynamic therapy (PDT) and immunotherapy has emerged as a promising strategy for treating solid tumors. This combination not only enhances local tumor ablation but also activates systemic antitumor immune responses. Polymer Nanoparticles, with their unique photodynamic properties and ability to integrate multiple therapeutic modalities, offer a powerful platform for photo-immunotherapy. This review systematically discusses recent advances in the design of polymer Nanoparticles and their synergistic mechanisms when combined with immunomodulatory agents such as Toll-like receptor (TLR) agonists, STING agonists, and immune checkpoint inhibitors (ICBs). Moreover, we highlight challenges faced in clinical translation and outline future perspectives for the development of these combination therapies.

## 1. Introduction

Solid tumors remain one of the leading causes of cancer-related mortality worldwide, with traditional therapies such as surgery, chemotherapy, and radiation often limited by issues such as tumor recurrence, drug resistance, and side effects on healthy tissues. In recent years, the development of more targeted and less invasive therapies has become a critical focus in oncology. Among the most promising of these strategies are PDT and immunotherapy, both of which have demonstrated significant potential in tumor treatment [1]. However, while each of these therapies has its own advantages, combining them offers a unique opportunity to overcome individual limitations and enhance therapeutic efficacy.

PDT utilizes light-activated photosensitizers that generate reactive oxygen species (ROS) upon exposure to light, leading to tumor cell destruction. PDT has shown high selectivity for cancer cells, minimizing damage to surrounding healthy tissue, and can be used for both localized and metastatic tumors [2,3,4]. However, its effectiveness is often limited by issues such as low tumor penetration, limited ROS generation, and the development of resistance over time. Recent advances in nanomedicine, particularly the use of polymer nanoparticles as carriers for photosensitizers, have aimed to address these limitations. Polymer nanoparticles offer several advantages, including controlled drug delivery, enhanced stability, and the ability to functionalize surfaces for specific tumor targeting [5,6].

In parallel, immunotherapy has transformed cancer treatment by mobilizing the host immune system to eliminate malignant cells [7]. ICBs, including PD−1/PD−L1 inhibitors, have shown remarkable efficacy across multiple cancer types [8]. However, therapeutic responses remain limited in immunologically “cold” tumors due to immunosuppressive microenvironments and insufficient immune priming [9]. Notably, PDT has been shown to induce immunogenic cell death (ICD), promoting antigen release and immune activation [10]. This complementary mechanism provides a strong rationale for integrating PDT with immunotherapy to potentiate systemic antitumor responses [11].

Recent reviews on the integration of PDT and immunotherapy have largely focused on nanoplatform classification and combinational therapeutic strategies [12]. While these studies provide valuable insights into delivery systems and immune modulation approaches, comparatively limited attention has been given to how the intrinsic molecular structure of photosensitizers governs photophysical behavior and subsequent immune activation.

In photo-immunotherapy, the photosensitizer serves as the fundamental functional component responsible for ROS generation and ICD induction. Rational molecular design is therefore critical for precise immune modulation.

Polymer nanoparticles should not be viewed merely as passive carriers, but rather as structurally programmable photodynamic materials [13,14,15]. Their polymer topology, main-chain architecture, and side-chain functionalization collectively regulate excited-state dynamics, ROS generation pathways, and ICD intensity, thereby influencing dendritic cell maturation, antigen presentation, and T-cell priming.

Despite rapid progress in polymeric photosensitizers, a systematic framework linking molecular design parameters to immunological outcomes remains insufficiently established. The intrinsic relationship between structural engineering and immune activation has yet to be fully integrated into a coherent structure–function–immunology paradigm [16].

Therefore, this review focuses on polymeric nanoparticles as active photodynamic agents for synergistic photo-immunotherapy of solid tumors. We first systematically discuss the structural design strategies of polymeric photosensitizers, including polymer topology and configuration, main-chain engineering, side-chain functionalization, and biocompatibility regulation. We then summarize their therapeutic applications in solid tumors, highlighting synergistic mechanisms with immunotherapy and addressing key translational challenges and future perspectives, with the aim of providing a rational design framework for precise immune modulation and improved therapeutic efficacy.

## 2. Structure Design of Polymer

The structural design of polymeric photosensitizers fundamentally governs their photophysical properties, self-assembly behavior, and biological performance in PDT. Through rational modulation of polymer topology, backbone engineering, side-chain functionalization, and biocompatibility, these systems enable precise control over ROS generation, targeting capability, and in vivo delivery, while also influencing the induction of ICD and the remodeling of the tumor immune microenvironment. These interconnected structural dimensions therefore provide the molecular and functional basis for the synergistic integration of PDT with immunotherapy, as further discussed in the subsequent sections.

### 2.1. Polymer Topology and Architecture

Polymeric photosensitizers can be categorized into linear, branched, and dendritic or star-shaped architectures, representing increasing degrees of branching, functional group density, and spatial control. Topology directly determines molecular packing, aggregation patterns, and surface functionality distribution, thereby linking photophysical processes to biological interactions.

Linear polymers consist of single unbranched chains, such as PEG, PLA, PLGA, or linear conjugated polymers including polythiophene derivatives [17]. Their structural simplicity enables precise control over molecular weight and chain length, resulting in excellent synthetic reproducibility. Self-assembly is primarily governed by amphiphilic balance and intermolecular interactions including π stacking and hydrogen bonding. While linear systems offer robust manufacturability, the limited number of terminal functional groups constrains multifunctional integration and high density bioconjugation.

Branched polymers introduce secondary chains or multi-arm configurations, such as branched PEI, hyperbranched polyesters, and side chain grafted conjugated polymers [18]. Increased branching enhances functional group density and modifies intermolecular packing through steric effects. However, branch distribution is typically statistical, and structural precision remains limited by polymerization strategies. Dendritic or star-shaped polymers, including PAMAM dendrimers [19], star-shaped PEG, and star-shaped PLGA [20], exhibit generation controlled, symmetric architectures with well-defined molecular dimensions and densely organized peripheral groups. Compared to linear polymers, branched systems not only enhance the loading capacity for photosensitizing moieties, targeting ligands, or immune molecules, but also influence aggregation behavior and ROS generation efficiency through steric effects. In highly ordered dendritic systems, spatial confinement regulates intermolecular distances and exciton coupling, thereby affecting triplet state formation and ROS yield [19,21,22]. Dense surface functionality further promotes multivalent membrane interactions, improves cellular internalization, and may modulate immune receptor activation [23]. Although synthetic complexity increases with architectural precision, the enhanced spatial control offers superior functional integration.

Beyond topology, backbone electronic structure represents another critical design dimension. Conjugated polymers composed of alternating single and double bonds form delocalized π electron systems, including polythiophene, polyfluorene, polyphenylene vinylene, and Donor–Acceptor (D–A) type polymers [24]. Extended conjugation facilitates efficient light harvesting, exciton migration, and energy transfer, providing intrinsic photosensitizing capability [25]. Integration of electronically active backbones with defined topological architectures enables synergistic optimization of spatial organization and excited state dynamics, ultimately enhancing photodynamic efficiency and immunotherapeutic potential.

### 2.2. Polymer Backbones’ Design Strategies

While polymer topology governs supramolecular organization and aggregation behavior, the intrinsic photophysical properties of polymeric photosensitizers are determined by backbone electronic structure. Therefore, backbone engineering serves as a complementary strategy for tuning light absorption, excited state dynamics, and ROS generation.

#### 2.2.1. D–A Type

The incorporation of D–A architectures represents one of the most established and widely adopted backbone engineering strategies for tuning the photophysical properties of Polymer Nanoparticles. Alternating electron-donating and electron-accepting units along the polymer backbone induces strong intramolecular charge transfer (ICT), which effectively narrows the singlet–triplet energy gap (ΔE_ST), promotes intersystem crossing (ISC), and enhances triplet-state population and ROS generation efficiency [26]. Moreover, pronounced ICT leads to spatial separation of excited-state electron density, favoring electron-transfer pathways responsible for Type I ROS generation, which is particularly advantageous under hypoxic tumor conditions [27].

Common donor units include arylamines such as triphenylamine, carbazole, and benzodithiophene, while acceptor units typically consist of cyano-substituted heterocycles, benzothiadiazole, diimide derivatives, and pyridinium or indolium moieties [28]. Nevertheless, the photophysical advantages afforded by single D–A architectures are inherently constrained by the delicate balance among D–A strength, backbone planarity, and aggregation behavior, motivating the development of more advanced structural designs.

To address these limitations, expanding acceptor dimensionality has emerged as an effective strategy. Zhang et al. reported a dual-acceptor backbone design in which two strong acceptor units were sequentially incorporated into a polymer containing an arylamine donor. This cascade acceptor configuration intensified ICT, increased HOMO–LUMO separation, and further reduced ΔE_ST, leading to more efficient ISC without the use of heavy atoms. Compared with single-acceptor analogues, the dual-acceptor system exhibited significantly enhanced ROS generation, highlighting acceptor dimensionality expansion as a viable approach for ISC amplification [29]. However, such architectures inevitably increase synthetic complexity and may pose challenges in batch reproducibility and structure–property predictability.

D–A interaction strength also plays a decisive role in governing excited-state kinetics and ROS selectivity. Yang et al. constructed a series of ionic photosensitizers by pairing a strong donor (DTP) with indolium acceptors. Progressive enhancement of D–A strength amplified ICT, reduced ΔE_ST to as low as 0.54 eV, and significantly increased ISC efficiency in the aggregated state. Notably, the dominant ROS pathway shifted from Type II to Type I, underscoring the critical role of D–A interaction strength in dictating energy-level alignment, excited-state dynamics, and ROS generation pathways [30].

**Figure 1:**
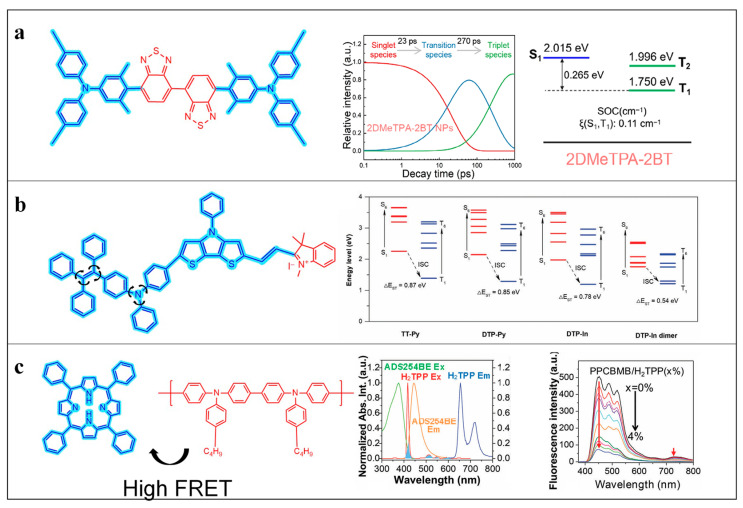
Structural design and photophysical mechanisms of representative D–A type photosensitizer systems. (**a**) D–A–A–D type compound 2DMeTPA-2BT exhibiting fast ISC, long triplet lifetime, and high triplet population [29]. Copyright © 2025 Wiley-VCH GmbH. (**b**) D–A type compound DTP-In showing pronounced spectral redshift and reduced singlet–triplet energy gap [30]. Copyright © 2024 Wiley-VCH GmbH. (**c**) Supramolecular D–A host–guest system composed of H_2_TPP and ADS254BE enabling efficient FRET-mediated energy transfer [31]. Copyright © 2023 Wiley-VCH GmbH. Reprinted with permission from ref. [30] 2025 Wiley-VCH GmbH. Reprinted with permission from ref. [31] 2024 Wiley-VCH GmbH. Reprinted with permission from ref. [32] 2023 Wiley-VCH GmbH.

Beyond covalent polymer backbones, D–A concepts have been extended to supramolecular assemblies. Zhao et al. reported a noncovalent D–A system formed via self-assembly between a conjugated polymer donor (ADS254BE) and a porphyrin acceptor (H_2_TPP), as summarized in Figure 1. The resulting fluorescence resonance energy transfer (FRET) pathway suppressed porphyrin self-quenching and enhanced ROS generation by optimizing excited-state energy transfer [31]. Although this approach demonstrates the general applicability of D–A principles, the dynamic nature of supramolecular assemblies may introduce variability under physiological conditions.

#### 2.2.2. AIE-Type

Under physiological conditions, polymer nanoparticles inevitably form nanoscale aggregates, rendering aggregation-state structure a decisive determinant of excited-state coupling and ROS generation efficiency. Backbone engineering has therefore been extensively employed to regulate intermolecular π–π interactions and aggregation modes (e.g., H- versus J-type aggregation), with the aim of reshaping excited-state dynamics and stabilizing triplet states [32]. However, aggregation effects are inherently dualistic and difficult to balance: while controlled aggregation can enhance photodynamic activity, excessive or poorly regulated aggregation frequently induces aggregation-caused quenching (ACQ), which remains a major bottleneck for efficient ROS generation in polymeric systems.

Rather than avoiding aggregation entirely, several studies have demonstrated that traditionally unfavorable H-aggregation can be rendered functionally productive through deliberate backbone constraints. For example, planar thiazine dyes such as Nile blue derivatives were covalently incorporated into polymer backbones to enforce ordered face-to-face stacking. This constrained H-aggregation suppressed disordered π–π interactions and significantly prolonged triplet lifetimes, resulting in approximately 3.0-fold and 2.9-fold increases in superoxide and singlet oxygen generation, respectively, compared with small-molecule counterparts [13]. Although effective, this strategy relies heavily on precise control of backbone planarity and packing order, which may limit its generalizability across diverse chromophore systems.

**Figure 2:**
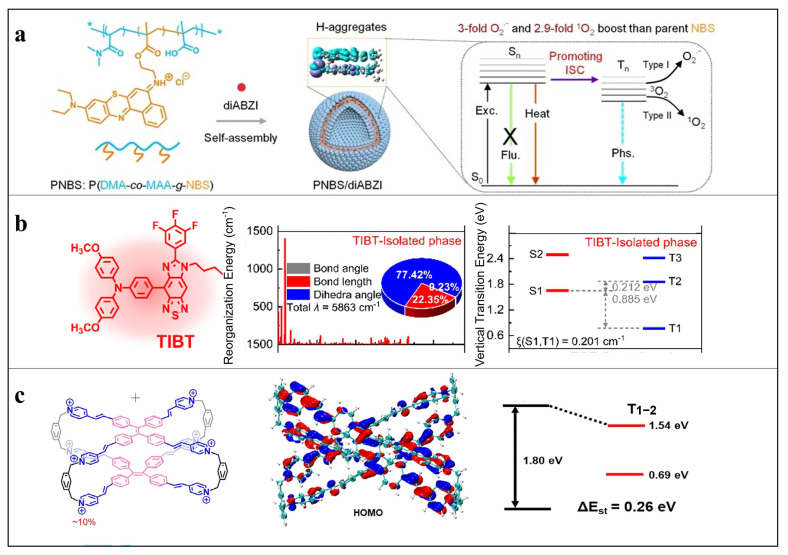
Structural design and photophysical mechanisms of representative AIE-type photosensitizer systems. (**a**) Schematic illustration of PNBS/diABZI nanovesicles, in which diABZI is encapsulated within the shell of polymersomes self-assembled from copolymers grafted with the Type I photosensitizer sulfur-substituted Nile Blue (NBS). PNBS/diABZI exhibits amplified generation of O_2_•^−^ and ^1^O_2_ due to enhanced intersystem crossing rates [13]. Copyright © 2024 American Chemical Society. (**b**) Structural features of TIBT nanocrystals (TIBT-NCs). In the crystalline aggregated state, ΔE_S1–T1 slightly decreases, while the spin–orbit coupling constant (ξ) is significantly enhanced to 0.398 cm^−1^, indicating accelerated ISC and more efficient ROS generation [37]. Copyright © 2024 Wiley-VCH GmbH. (**c**) Structure of the supramolecular complex 1@CB [8]_4_ and calculated HOMO–LUMO distributions and energy levels, revealing reduced ΔE_ST and improved orbital separation, which facilitate electron transfer to molecular oxygen and enhanced O_2_•^−^ generation. Here, T_1_ and T_2_ refer to the first and second excited triplet states, respectively, and T_1_–_2_ denotes the energy gap between them. T_2_ energy levels (1.12–1.54 eV) exceed the oxygen sensitization threshold (0.98 eV), resulting in comparable ^1^O_2_ generation efficiencies [36]. Copyright © 2024 SCUT, AIEI, and John Wiley & Sons Australia, Ltd. Reprinted with permission from ref. [13] 2024 Chemical Society. Reprinted with permission from ref. [38] 2024 Wiley-VCH GmbH. Reprinted with permission from ref. [37] 2024 SCUT, AIEI, and John Wiley & Sons Australia, Ltd.

To more fundamentally address ACQ, aggregation-induced emission (AIE) mechanisms have been increasingly integrated into backbone design [33]. Twisted structural motifs, such as tetraphenylethylene (TPE) and triphenylamine (TPA), introduce steric hindrance that restricts intramolecular motion in the aggregated state, thereby suppressing nonradiative decay and stabilizing excited states [34]. The restricted intramolecular rotation (RIR) mechanism has been widely recognized as a key physical origin of sustained ROS generation in AIE-type photosensitizers and is closely associated with enhanced induction of ICD [35]. Nevertheless, single-level RIR regulation may be insufficient under complex biological conditions. Recent advances have therefore combined covalent backbone constraints with noncovalent supramolecular interactions to achieve multilevel RIR regulation. In one representative study, TPA-based chromophores were covalently embedded into conjugated backbones and further immobilized through supramolecular assembly, enabling dual RIR control. This synergistic restriction more effectively suppressed nonradiative decay, prolonged excited-state lifetimes, and substantially enhanced ROS generation [36]. Beyond rigidity, backbone asymmetry has emerged as an additional yet underexplored parameter. Zhao et al. demonstrated that asymmetric D–A photosensitizers formed dynamic and reversible aggregates in aqueous environments; compared with symmetric analogues, these structures weakened excessive π–π stacking, mitigated ACQ, and preserved long-lived triplet states, thereby enabling sustained ROS generation and prolonged PDT efficacy [37]. Together, these aggregation-regulation strategies and multilevel structural designs are schematically illustrated in Figure 2.

#### 2.2.3. Conformational Isomerism

Beyond electronic structure modulation, backbone conformation represents a critical yet often underappreciated determinant of aggregate-state photophysics in polymeric photosensitizers. Highly planar and rigid backbones can enhance π-conjugation, light absorption, and charge delocalization, but frequently exacerbate intermolecular π–π stacking in the aggregated state, leading to accelerated nonradiative decay, triplet quenching, and diminished ROS productivity [38,39]. This intrinsic trade-off underscores the importance of conformational regulation rather than indiscriminate conjugation extension.

Steric perturbation via bulky substituents, noncoplanar linkages, or conformationally flexible segments provides an effective route to balance conjugation efficiency with excited-state stability. These strategies modulate ICT, ISC efficiency, ΔE_ST, and aggregation modes, collectively reshaping ROS generation pathways [40]. Twisted units, flexible linkers, or alternating rigid–flexible backbone motifs can alleviate over-conjugation and excessive interchain coupling, thereby stabilizing triplet states. In this regard, backbone conformation serves as an intermediate regulatory layer bridging molecular electronic structure and macroscopic photodynamic performance.

Huang et al. exemplified this principle by constructing Donor–π–Acceptor (D–π–A) backbones to develop AIE-type ionic photosensitizers (BuDTTPy). Through systematic tuning of donor strength and steric bulk using pyridinium acceptors, they enhanced ICT while maintaining a twisted molecular conformation. The bulky alkyl-substituted BuDTTPy exhibited reduced ΔE_ST and strengthened spin–orbit coupling, favoring Type I ROS generation even under hypoxic conditions and enabling NIR-II fluorescence imaging-guided PDT [41]. It should be noted, however, that excessive conformational distortion may impede charge mobility and long-range exciton diffusion, necessitating careful structural optimization. Beyond local conformational control, backbone topology and spatial confinement further influence aggregate-state photophysics [42]. Although effective, the synthetic complexity and limited tunability of porous frameworks may restrict broader applicability.

### 2.3. Polymer Side-Chains’ Design Strategies

Side-chain functionalization is a central strategy for enhancing the therapeutic performance of polymeric photosensitizers in combined photodynamic and immunotherapy. By introducing specific chemical groups into side chains, polymers can achieve targeting capability, stimulus-responsiveness, and hydrophilic/hydrophobic balance, which together improve water solubility, colloidal stability, and modulate self-assembly behavior. These modifications not only optimize the local chemical environment around the polymer backbone but also enhance intracellular ROS production, particularly under hypoxic or acidic tumor microenvironments, providing a solid foundation for effective photodynamic and immune responses [43].

#### 2.3.1. Targeting Element Modification

To improve tumor selectivity and intracellular accumulation, side chains can be modified with targeting ligands such as RGD peptides, folic acid, saccharides, or antibody fragments. These ligands enable polymeric nano-photosensitizers to recognize receptors overexpressed on tumor cells or tumor vasculature, facilitating receptor-mediated endocytosis [44,45,46,47]. Beyond cellular targeting, organelle-specific groups—such as mitochondria-targeting triphenylphosphonium or lysosome-targeting morpholine—can further direct ROS generation to critical subcellular sites, promoting oxidative damage, damage-associated molecular patterns (DAMPs) release, and ICD while minimizing off-target phototoxicity [48,49]. This precise localization also establishes a foundation for subsequent stimulus-responsive activation.

#### 2.3.2. Stimulus-Responsive Units

Tumor microenvironments typically exhibit mild acidity, elevated intracellular glutathione, and abnormal enzyme expression, providing ideal triggers for stimulus-responsive side chains [45]. Acid-labile linkers, disulfide bonds, or enzyme-cleavable peptide sequences enable selective activation or release of photosensitizers and immunomodulators at the tumor site [50,51,52]. By integrating these responsive units with targeting modifications, polymers can achieve spatiotemporal control over ROS production and immune activation, maximizing therapeutic efficacy while minimizing systemic toxicity.

#### 2.3.3. Stereospecific Blockade and Self-Assembly

**Figure 3:**
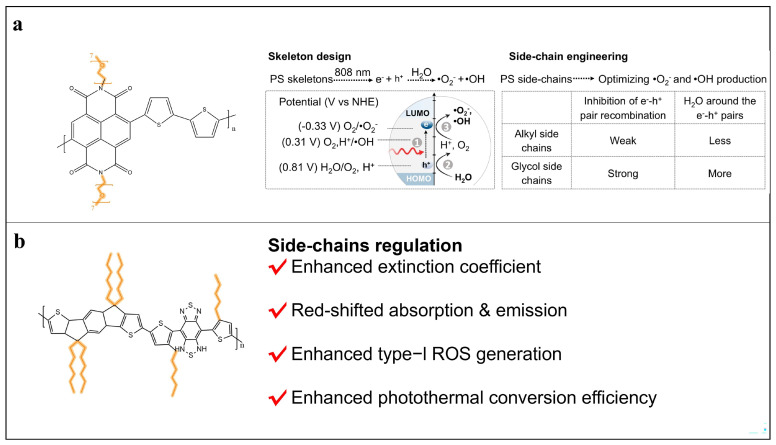
Structural design and photophysical mechanisms of representative stereospecific-blockade photosensitizer systems. (**a**) Chemical structures of NTgly polymers featuring glycol side chains that increase permittivity and hydrophilicity, suppress electron–hole recombination, and enhance •O_2_^−^ and •OH generation [53]. Copyright 2024, Springer Nature. (**b**) Structure of PIDT(He)TBT with a D–A type conjugated backbone, in which flexible side chains and high molecular weight confer enhanced AIE properties, red-shifted absorption/emission, photostability, and Type I PDT performance [55]. In this figure, the colored highlights and arrows denote the functional side chains described in the main text. These side chains are critically involved in stereospecific blockade and self-assembly. Copyright © 2025 American Chemical Society. Reprinted with permission from ref. [54] 2024 Springer Nature. Reprinted with permission from ref. [56] 2025 American Chemical Society.

Side-chain engineering has emerged as a versatile strategy to regulate both the molecular and supramolecular behaviors of polymeric photosensitizers. At the molecular level, stereospecific blockade using branched, bulky, or flexible side chains can precisely tune interchain spacing, reshape aggregation microstructures, and control excited-state dynamics, maintaining strong light absorption while enhancing ROS stability. Tang et al. demonstrated that OEG side chains in naphthalimide–bithiophene polymers increased dielectric constant, suppressed charge recombination, and enriched water as a reactive substrate, resulting in ~4-fold higher •O_2_^−^ and up to 51-fold higher •OH generation compared with methylene blue [53]. Similarly, Chen et al. reported that diisopropylethylamine donor side chains in polymer-brush BODIPY photosensitizers stabilized charge-separated states and favored Type I ROS generation under hypoxia [54], while flexible aliphatic side chains in IDT–BBT backbones enhanced backbone planarity, ICT strength, NIR-II absorption, and triplet lifetimes, further improving Type I ROS production [55]. Representative stereospecific-blockade systems are summarized in Figure 3.

These molecular-level modifications significantly enhance the water solubility and self-assembly behavior of polymeric photosensitizers, offering distinct advantages over conventional small-molecule photosensitizers, which are often hydrophobic, prone to aggregation in aqueous environments, and consequently exhibit reduced ROS generation and limited bioavailability [4].

In contrast, polymeric photosensitizers incorporate abundant hydrophilic side chains, such as PEG [56], OEG [57], or charged/polar groups, which form hydration shells around the macromolecule and enhance dispersibility. Additionally, the cumulative effect of hydrophilic repeating units within the polymer backbone further improves overall solubility and enables stable self-assembly into nanoparticles. This self-assembly not only improves colloidal stability and intracellular delivery but also reduces nonspecific protein adsorption, prolongs circulation, and promotes EPR-mediated tumor accumulation [58,59]. By combining steric effects with amphiphilic or hydrophobic segments, such as alkyl chains or aromatic units, polymers can form well-defined core–shell nanostructures, allowing simultaneous optimization of nanoparticle morphology, ROS generation efficiency, and immunogenic signaling. Overall, rational side-chain design that integrates steric, hydrophilic, and amphiphilic features provides a coherent strategy to maximize water solubility, ROS production, and the synergistic photodynamic and immunotherapeutic efficacy of polymeric photosensitizers.

### 2.4. Heavy Metal Coordination

Incorporation of metal components represents a powerful yet nontrivial strategy to enhance ISC, diversify ROS pathways, and expand functionality. In polymeric systems, metal centers act cooperatively with organic frameworks rather than as independent photoactive units [60,61]. Heavy-metal-induced spin–orbit coupling effectively compensates for limited ISC efficiency in purely organic polymers. Transition-metal complexes such as Ir(III), Ru(II), and Pt(II) have been incorporated into polymer backbones or side chains to elevate triplet populations and ROS yields [62,63,64]. However, excessive metal loading may introduce nonradiative decay or destabilize colloidal systems. Beyond ISC enhancement, metal coordination enables multimodal therapy and immune activation through amplified ROS-induced ICD.

Beyond ISC enhancement, metal–polymer cooperative systems offer opportunities to extend polymeric photosensitizers beyond single-modality PDT. Certain metal centers endow additional functionalities, including photothermal conversion, imaging contrast, or radio-sensitization, thereby enabling synergistic therapeutic modalities within a single platform. From an immunological perspective, metal-amplified ROS production can facilitate the release of DAMPs and promote ICD, generating a tumor immune microenvironment more amenable to combination with immune checkpoint blockade or immunostimulatory agents.

**Figure 4:**
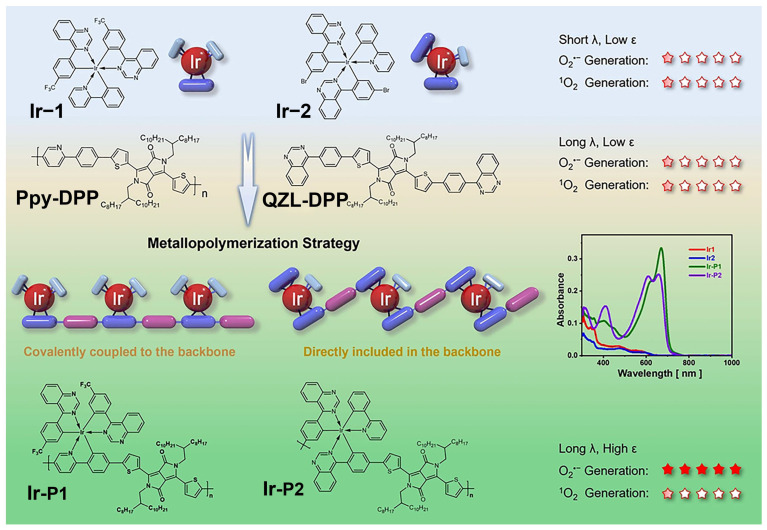
Structural design and photophysical mechanisms of representative heavy-metal-coordinated polymeric photosensitizers based on Ir(III) complexes, illustrating ISC enhancement and Type I ROS generation [65]. Copyright 2024, Springer Nature. Reprinted with permission from ref. [66] 2024 Springer Nature.

A representative example involves Ir(III)-embedded conjugated metallopolymers, where robust Ir–C bonds enhance SOC while polymer backbones ensure strong NIR absorption. These systems favor electron-transfer-driven O_2_•^−^ and •OH generation under hypoxia and induce potent ICD [65]. These metal–polymer cooperative mechanisms and their immunological implications are schematically illustrated in Figure 4.

### 2.5. Biocompatibility

Natural polymers possess inherent biocompatibility and biodegradability, making them attractive carriers for photosensitizers [66]. However, their limited chemical tunability and functionalization capacity restrict precise control over photophysical properties, water solubility, and pharmacokinetics.

In contrast, synthetic polymers offer precise structural control, tunable chemical composition, and versatile functionalization, enabling optimization of solubility, stability, and targeting capability. Common synthetic polymers include PEG, PLA, PLGA and polypeptides [67]. Through rational design, these polymers can achieve good water solubility and in vivo stability while being gradually cleared via biodegradation or metabolic pathways. For example, PLA and PLGA degrade into lactic and glycolic acids, which enter natural metabolic routes, whereas PEG-modified polymers can be slowly eliminated through renal excretion [68].

The combination of controllable degradability, tunable hydrophilicity, and predictable pharmacokinetics allows synthetic polymers to balance therapeutic efficacy with safety. Moreover, these features provide opportunities for functional modifications that enhance tumor targeting, intracellular delivery, and ROS generation efficiency, making them highly suitable for photodynamic and immunotherapeutic applications.

## 3. Therapeutic Application of Polymer Nanoparticles in Solid Tumors

**Figure 5:**
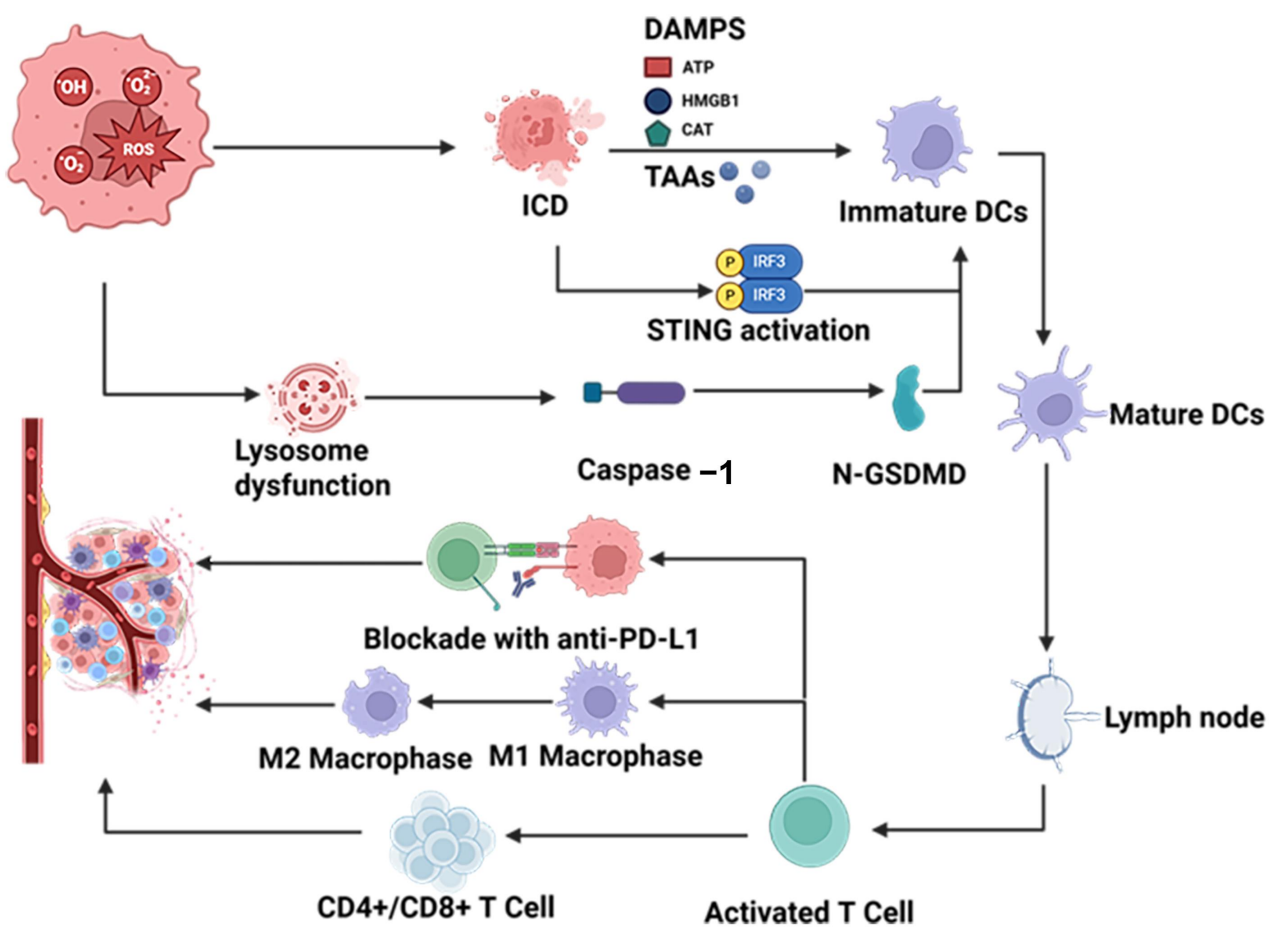
Mechanistic illustration of Polymer nanoparticle-mediated PDT synergized with immunotherapy for solid tumor treatment.

Polymer nanoparticles enhance immunotherapy by inducing ICD and reshaping the tumor immune microenvironment. Upon irradiation, PDT-generated ROS promote tumor-associated antigens (TAAs) and DAMPs release, converting “cold” tumors into “hot” tumors and sensitizing them to immunotherapy. These coordinated immunomodulatory processes form the mechanistic basis of Polymer nanoparticle-mediated PDT synergized with immunotherapy, as illustrated in Figure 5.

### 3.1. Polymer Nanoparticles Loading with TLR Agonists

TLRs are a conserved family of pattern-recognition receptors that serve as central sensors of pathogen-associated molecular patterns (PAMPs) and DAMPs in innate immunity. To date, ten human TLRs (TLR1–TLR10) have been identified, with broad expression on antigen-presenting cells (APCs) such as dendritic cells (DCs) and macrophages, as well as on subsets of adaptive immune cells [69,70]. Upon activation, TLR signaling induces the production of pro-inflammatory cytokines and type I interferons (IFNs), while simultaneously promoting APC maturation and antigen presentation. This dual functionality positions TLRs as critical molecular bridges between innate and adaptive antitumor immunity [71].

Polymer nanoparticle-mediated PDT provides a natural upstream trigger for TLR signaling by inducing ICD and releasing DAMPs such as calreticulin (CRT), HMGB1, and ATP [72]. These signals facilitate antigen uptake and presentation by DCs, ultimately promoting tumor-specific T-cell activation and CD8^+^ T-cell infiltration [73,74]. However, accumulating evidence indicates that PDT-induced ICD alone often fails to elicit sufficiently strong or durable immune responses, largely due to limited APC activation efficiency and inadequate inflammatory amplification. In this context, exogenous TLR agonists serve as rational immunological amplifiers.

TLR agonists enhance PDT efficacy through two complementary mechanisms: first, by directly promoting DC maturation and antigen-presenting capacity; second, by synergistically intensifying PDT-induced ICD. For example, the TLR7/8 agonist imiquimod (IMQ) not only activates DCs and induces IL-12 and IFN-γ secretion, but also significantly enhances PDT-induced CRT exposure, HMGB1 release, and ATP secretion, thereby strengthening tumor immunogenicity [75,76,77]. In addition, TLR agonists actively remodel the immunosuppressive tumor microenvironment (TME) by reprogramming tumor-associated macrophages (TAMs) from the M2 to the M1 phenotype, reducing immunosuppressive cytokine secretion and enhancing antigen presentation [78,79,80,81,82,83,84,85,86].

**Figure 6:**
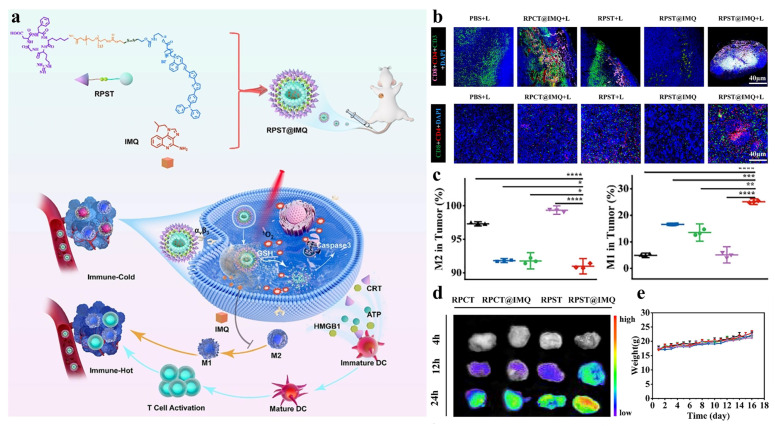
Schematic illustration and immunological evaluation of RPST@IMQ-mediated photo-immunotherapy via TAM reprogramming [15]. (**a**) Schematic illustration of the preparation and mechanism of RPST@IMQ for photo-induced immunotherapy through TAM reprogramming. (**b**) Representative immunofluorescence images showing T lymphocytes in tumor tissue of C57BL/6 tumor-bearing mice (pink: CD8^+^; red: CD4^+^; green: CD3^+^). Representative images of CD4^+^ (red) and CD8^+^ (green) T cells in spleen tissue. (**c**) Quantitative analysis of M1 (F4/80^+^ CD86^+^) and M2 (F4/80^+^ CD206^+^) macrophages in tumor tissues from mice subjected to various treatments. (**d**) Fluorescence imaging of mouse tumors at 4, 12, and 24 h post-intravenous administration of prodrugs. (**e**) Changes in tumor volume over time after various treatments. Copyright © 2025 SCUT, AIEI, and John Wiley & Sons Australia, Ltd. Reprinted with permission from ref. [91] 2025 SCUT, AIEI, and John Wiley & Sons Australia, Ltd.

Despite these advantages, systemic administration of TLR agonists remains clinically problematic due to poor solubility, rapid clearance, insufficient tumor accumulation, and dose-limiting immune toxicity [87,88,89]. Polymer nanoparticle-based delivery systems offer an effective solution by enabling tumor-targeted accumulation, controlled release, and synchronized activation with PDT. For instance, Zhang et al. developed a cRGD-modified nanoplatform (RPST@IMQ) that achieves integrin αvβ3–mediated tumor targeting. Upon light irradiation, PDT induces ICD, while elevated intratumoral glutathione triggers disulfide bond cleavage and IMQ release. This coordinated activation significantly increased the intratumoral M1/M2 TAM ratio and enhanced CD8^+^ T-cell infiltration by 1.88-fold compared with PDT alone, resulting in potent suppression of both primary and metastatic tumors [15]. Figure 6 schematically illustrates this RPST@IMQ-mediated photo-immunotherapy strategy, highlighting both the TAM reprogramming and the enhanced antitumor immune responses.

Similarly, CpG oligodeoxynucleotides (CpG-ODNs), as TLR9 agonists, have been extensively combined with polymer nanoparticles to enhance DC maturation and T-cell activation [90]. Zhong et al. reported a dendritic polymer nanoplatform (G5-HP/CpG) that integrates PDT, photothermal therapy, and TLR9 activation. This multimodal system achieved superior DC maturation, enhanced effector T-cell infiltration, and prolonged survival in tumor-bearing mice compared with monotherapies [91].

Collectively, these studies demonstrate that Polymer nanoparticle-enabled delivery of TLR agonists not only overcomes pharmacokinetic and toxicity limitations but also functionally couples ICD induction with innate immune activation, thereby achieving immune responses unattainable by either modality alone.

### 3.2. Polymer Nanoparticles Loading with STING Agonists

STING agonists comprise a class of small-molecule or biological agents designed to activate the STING pathway, a central innate immune signaling axis that functionally links cytosolic danger sensing to adaptive antitumor immunity [92,93,94,95]. Under physiological conditions, the STING pathway is activated by cytosolic double-stranded DNA derived from pathogens or damaged tumor cells. This DNA is sensed by cyclic GMP–AMP synthase (cGAS), which catalyzes the production of the second messenger cyclic GMP–AMP (cGAMP). CGAMP subsequently binds to STING on the endoplasmic reticulum membrane, triggering STING translocation and activation of the downstream TBK1–IRF3 signaling cascade. Phosphorylated IRF3 then translocates to the nucleus, driving the transcription of type I IFNs and a broad spectrum of pro-inflammatory cytokines [96,97,98]. The secretion of type I IFNs and inflammatory mediators plays a decisive role in antitumor immunity by promoting DC maturation, enhancing antigen processing and presentation, and facilitating the recruitment and activation of CD8^+^ cytotoxic T lymphocytes. Through this mechanism, STING activation reshapes the TME, alleviates local immunosuppression, and converts immunologically “cold” tumors into “hot” tumors characterized by robust immune cell infiltration and responsiveness to immunotherapy. Importantly, PDT provides a mechanistically complementary upstream trigger for STING activation. PDT-induced ROS cause ICD and extensive tumor cell damage, leading to the release of tumor-derived DNA into the cytosol. This DNA serves as an endogenous ligand for cGAS, thereby priming the cGAS–STING pathway. However, PDT alone often induces insufficient or transient STING activation due to limited cytosolic DNA accumulation and rapid signal attenuation. The introduction of exogenous STING agonists therefore acts as a critical amplification step, ensuring robust and sustained activation of the STING–TBK1–IRF3 axis in the context of PDT-induced ICD.

Despite their strong immunostimulatory potential, the clinical translation of STING agonists remains constrained by poor in vivo stability, low cellular uptake efficiency, and the risk of systemic immune toxicity resulting from uncontrolled cytokine release [99,100]. Polymer nanoparticle-based delivery strategies offer an effective solution to these challenges by enabling tumor-targeted accumulation, spatiotemporally controlled release, and synchronization of STING activation with PDT-induced ICD.

**Figure 7:**
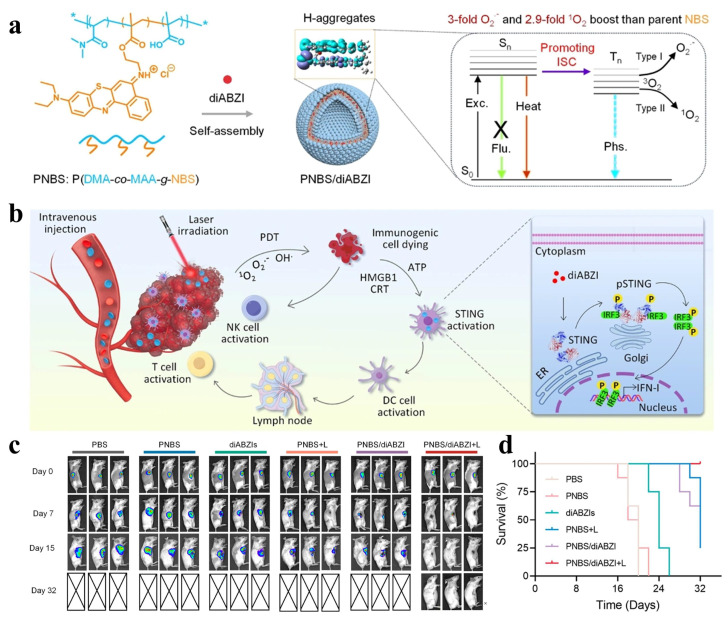
Structural design and therapeutic performance of PNBS/diABZI nanovesicles for PDT-amplified STING immunotherapy [13]. (**a**) Schematic illustration of PNBS/dIABZI nanovesicles. The STING agonist diABZI is encapsulated within the shell of polymersomes assembled from copolymers grafted with the Type I photosensitizer sulfur-substituted Nile Blue (NBS). The production of superoxide anion (O_2_•^−^) and singlet oxygen (^1^O_2_) by PNBS/dIABZI is amplified 3-fold and 2.9-fold, respectively, compared to the parent photosensitizer NBS, owing to enhanced intersystem crossing rates. (**b**) PNBS/dIABZI nanovesicles potentiate STING-mediated immunotherapy. (**c**) Whole-animal in vivo bioluminescence imaging of 4T1-luci breast tumor-bearing mice after various treatments. The experiment was performed twice with similar results. (**d**) Survival curves of mice after treatment (n = 8 biologically independent animals per group). Copyright © 2024 American Chemical Society. Reprinted with permission from ref. [13] 2024 American Chemical Society.

Representative examples illustrate the advantages of this integrated strategy. Wang et al. developed a PNBS/diABZI nanoplatform in which the STING agonist diABZI is encapsulated within polymeric nanocarriers, as shown in Figure 7. Upon light irradiation, Polymer nanoparticle-mediated PDT induces ICD, while simultaneously enabling in situ release of diABZI, resulting in robust activation of the STING–IRF3 pathway and elevated IFN-β secretion. In a 4T1 breast cancer mouse model, this coordinated activation achieved complete tumor eradication, underscoring the therapeutic benefit of coupling PDT with STING signaling through polymer nanoparticles [13]. Similarly, An et al. reported a protease B-responsive STING agonist prodrug (SAPCon) that achieves localized STING activation within the tumor without the need for an external carrier. This strategy effectively minimizes systemic exposure while maintaining potent antitumor efficacy, demonstrating improved safety and therapeutic performance in breast cancer models [101].

In summary, STING agonists are powerful immunotherapeutic agents that bridge innate and adaptive immunity through type I IFN-dominated signaling. However, their clinical application is constrained by delivery inefficiency and systemic toxicity. Integration with Polymer nanoparticle-mediated PDT provides a mechanistically rational solution by synchronizing ICD-induced danger signaling with localized STING activation. This combinatorial strategy amplifies antitumor immune responses, promotes favorable remodeling of the tumor microenvironment, and improves the safety profile of STING agonists, underscoring the potential of polymer nanoparticle–STING platforms for next-generation photo-immunotherapeutic applications.

### 3.3. Polymer Nanoparticles Loading with ICBs

Immune checkpoints, including PD−1/PD-L1 and CTLA−4, are key regulators of tumor immune evasion [102]. Engagement of PD−1 on activated T cells with PD-L1 expressed by tumor cells or APCs inhibits T-cell proliferation, cytokine production, and cytotoxic activity, whereas CTLA−4 attenuates T-cell priming by competing with CD28 for binding to CD80/CD86 on DCs, thereby weakening the initial activation signal [103,104,105,106]. By blocking these inhibitory pathways, ICBs can restore the antitumor function of T cells [107,108,109]. However, the therapeutic efficacy of ICBs is highly dependent on pre-existing tumor immunogenicity, particularly sufficient tumor antigen availability and effective T-cell infiltration within the tumor microenvironment. In immunologically “cold” tumors, characterized by limited antigen release, inefficient antigen presentation, and poor effector T-cell recruitment, ICB monotherapy often fails to elicit durable responses.

Polymeric nanoparticles provide a mechanistically complementary strategy to overcome these limitations by combining PDT with ICBs. Upon light irradiation, Polymer nanoparticle-mediated PDT induces ICD, leading to the release of TAAs and DAMPs, which promote dendritic cell maturation and efficient T-cell priming. Simultaneously, PDT-generated ROS and inflammatory cytokines activate tumor stress-response pathways, including JAK/STAT and NF-κB signaling, often resulting in compensatory upregulation of immune inhibitory molecules such as PD−L1 on tumor cells [110,111,112,113,114,115]. This dual effect—immune activation coupled with adaptive checkpoint upregulation—provides a strong mechanistic rationale for combining PDT with ICBs.

Consistent with this rationale, numerous preclinical studies have demonstrated that polymer nanoparticles co-delivering photosensitizers and ICB-related agents (e.g., siPD-L1 or PROTAC-based inhibitors) enhance antitumor immunity, increase CD8^+^ T-cell infiltration, repolarize TAM toward an antitumoral M1 phenotype, and suppress solid tumor growth [16,116]. Translating these mechanistic insights into clinical settings, recent studies have begun investigating PDT in combination with ICBs for human solid tumors. For example, the open-label study NCT04305795 evaluates ASP-1929 photoimmunotherapy combined with anti-PD−1 therapy in recurrent or metastatic head and neck squamous cell carcinoma. A phase III trial (NCT06699212) compares ASP-1929 plus pembrolizumab with standard pembrolizumab therapy in locoregionally recurrent HNSCC, while a phase II study (NCT06381154) assesses Verteporfin-mediated PDT in combination with pembrolizumab and standard chemotherapy in advanced or metastatic gastric/gastroesophageal junction cancer. Additionally, a phase I b study combining ASP-1929 photoimmunotherapy with nivolumab has demonstrated safety and feasibility in patients with advanced gastric cancer. These clinical studies highlight the translational potential of PDT-induced ICD and immune modulation to sensitize solid tumors to ICBs.

Overall, the combination of polymer nanoparticle-mediated PDT with ICBs addresses the fundamental limitations of ICB monotherapy by converting immunologically “cold” tumors into “hot” tumors through ICD-driven antigen release, DC activation, and T-cell recruitment. Meanwhile, PDT-induced stress signaling promotes immune checkpoint upregulation, further reinforcing the mechanistic synergy between PDT and ICBs. Beyond ICBs and TLR agonists, polymer nanoparticles have also been integrated with metabolic modulators and TME-targeting regulators to alleviate additional immunosuppressive barriers, as summarized in Table 1 [117,118].

## 4. Challenges and Future Perspectives

In summary, polymer nanoparticles have emerged as a highly adaptable and mechanistically powerful platform for the integration of PDT with cancer immunotherapy. Through rational molecular and supramolecular design, polymeric photosensitizers enable precise regulation of excited-state dynamics, ROS generation pathways, and tumor-specific delivery behaviors, thereby addressing several intrinsic limitations of conventional small-molecule photosensitizers. Backbone engineering strategies, including D—A architectures, heteroatom incorporation, and conjugation modulation, establish the fundamental photophysical framework for efficient intersystem crossing and Type I/Type II ROS production, while side-chain functionalization and metal-coordination approaches ensure effective biological performance within complex tumor microenvironments.

**Figure 8:**
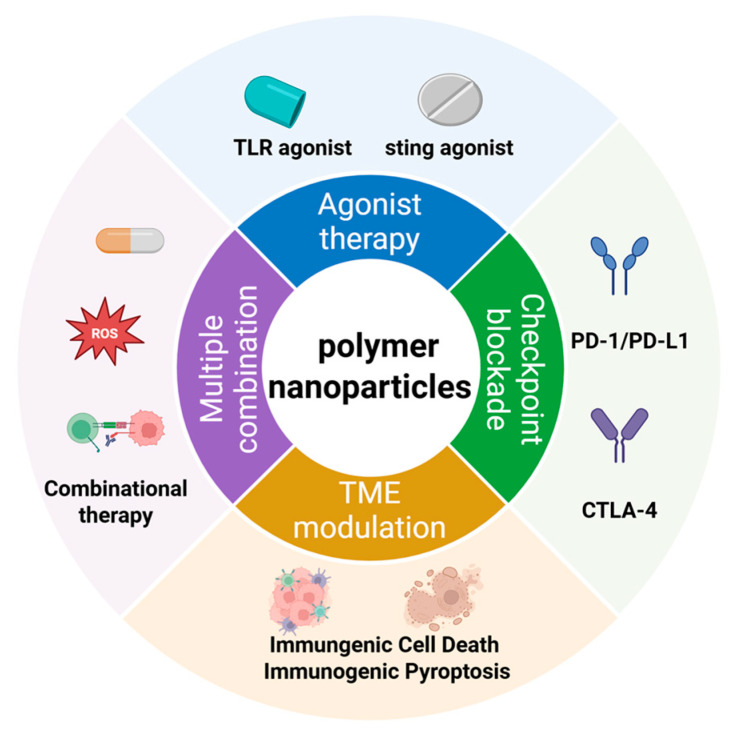
Combinatorial strategies of polymer nanoparticles with immunotherapy.

Importantly, polymer nanoparticle-mediated PDT functions not only as a localized cytotoxic modality but also as a potent immunological trigger. By inducing ICD, promoting tumor antigen release, and remodeling the immunosuppressive tumor microenvironment, polymer nanoparticles create a favorable context for synergistic combination with diverse immunomodulatory agents, such as TLR agonists, STING agonists, andICBs (Figure 8). The co-integration of photodynamic and immunotherapeutic functions within a single nanosystem enables spatiotemporally controlled immune activation and amplifies systemic antitumor immune responses beyond the irradiated tumor site.

Despite these promising advances, several challenges must be addressed before the clinical translation of polymer nanoparticle-based photo-immunotherapy can be realized. These include the scalable synthesis and batch-to-batch reproducibility of structurally complex polymers, precise control over in vivo pharmacokinetics and biodegradability, and a deeper mechanistic understanding of how polymer structure governs immune activation at the molecular and cellular levels. Moreover, standardized evaluation models that more accurately recapitulate human tumor immunology are essential for bridging the gap between preclinical efficacy and clinical outcomes.

Looking forward, continued progress in polymer chemistry, photophysics, and tumor immunology is expected to drive the development of next-generation polymer nanoparticleswith enhanced therapeutic precision and translational potential. By uniting molecular-level design with immunological function, polymeric photosensitizers are poised to play an increasingly important role in the advancement of synergistic photo-immunotherapeutic strategies for solid tumor treatment.

## Figures and Tables

**Table 1:** Summary of polymer nanoparticle-based combination strategies with immunotherapeutic modalities.

Polymer Nanoparticle	Drug	Therapy	Ref.
SPNE	DOX, R848	PDT/Immune Agonists	[119]
BMR	Mel, R848	PDT/Immune Agonists	[120]
SPNK	KYNase	PDT/Metabolic Modulators	[121]
SPH_9a_	Gox, ADA	PDT/Metabolic Modulators	[122]
SPDMCN	DMC	PDT/TME Modulators	[123]
PSP Bodipy	Reg	PDT/Immune Agonists/TME Modulators	[124]
THPP	VK3, WP1066	PDT/Immune Agonists/TME Modulators	[118]
SPNcb	BAPN, aPD-L1	PDT/Metabolic Modulators/ ICBs	[125]
PAP	MEL, SRF, α-PD1	PDT/PTT/ICBs/TME Modulators	[126]
SPNe	Sitagliptin, aCTLA-4	PDT/Metabolic Modulators/ICBs	[127]

## Data Availability

No new data were created or analyzed in this study. Data sharing is not applicable to this article.

## Abbreviations

The following abbreviations are used in this manuscript:

ACQ	Aggregation-caused quenching
AIE	Aggregation-induced emission
APCs	Antigen-presenting cells
CRT	Calreticulin
DAMPs	Damage-associated molecular patterns
DCs	Immunogenic cell death
D–A	Donor–Acceptor
EPR	Enhanced permeability and retention effect
FRET	Fluorescence resonance energy transfer
IFNs	Interferons
ICD	Immunogenic cell death
ICBs	Immune checkpoint inhibitors
IMQ	Imiquimod
ISC	Intersystem crossing
ICT	Intramolecular charge transfer
PAMPs	Pathogen-associated molecular patterns
PDT	Photodynamic therapy
ROS	Reactive oxygen species
RIR	Restricted intramolecular rotation
TLR	Toll-like receptor
TAAs	Tumor-associated antigens
TAMs	Tumor-associated macrophages
TME	Tumor microenvironment
TPE	Tetraphenylethylene
TPA	Triphenylamine
ΔE_ST	The singlet–triplet energy gap
